# Assimilation of SMAP Brightness Temperature Observations in the GEOS Land–Atmosphere Data Assimilation System

**DOI:** 10.1109/jstars.2021.3118595

**Published:** 2021-10-07

**Authors:** Rolf H. Reichle, Sara Q. Zhang, Qing Liu, Clara S. Draper, Jana Kolassa, Ricardo Todling

**Affiliations:** Global Modeling and Assimilation Office, NASA Goddard Space Flight Center, Greenbelt, MD 20771 USA; Global Modeling and Assimilation Office, NASA Goddard Space Flight Center, Greenbelt, MD 20771 USA,; Science Applications International Corporation, Reston, VA 20190 USA; Global Modeling and Assimilation Office, NASA Goddard Space Flight Center, Greenbelt, MD 20771 USA,; Science Systems and Applications, Inc., Lanham, MD 20771 USA; Physical Sciences Laboratory, NOAA Earth System Research Laboratories, Boulder, CO 80305 USA; Global Modeling and Assimilation Office, NASA Goddard Space Flight Center, Greenbelt, MD 20771 USA,; Science Systems and Applications, Inc., Lanham, MD 20771 USA; Global Modeling and Assimilation Office, NASA Goddard Space Flight Center, Greenbelt, MD 20771 USA

**Keywords:** Data assimilation, microwave remote sensing, soil moisture, soil moisture active passive (SMAP)

## Abstract

Errors in soil moisture adversely impact the modeling of land–atmosphere water and energy fluxes and, consequently, near-surface atmospheric conditions in atmospheric data assimilation systems (ADAS). To mitigate such errors, a land surface analysis is included in many such systems, although not yet in the currently operational NASA Goddard Earth Observing System (GEOS) ADAS. This article investigates the assimilation of L-band brightness temperature (Tb) observations from the Soil Moisture Active Passive (SMAP) mission in the GEOS weakly coupled land–atmosphere data assimilation system (LADAS) during boreal summer 2017. The SMAP Tb analysis improves the correlation of LADAS surface and root-zone soil moisture versus *in situ* measurements by ~0.1–0.26 over that of ADAS estimates; the unbiased root-mean-square error of LADAS soil moisture is reduced by 0.002–0.008 m^3^/m^3^ from that of ADAS. Furthermore, the global land average RMSE versus *in situ* measurements of screen-level air specific humidity (q2m) and daily maximum temperature (T2m_max_) is reduced by 0.05 g/kg and 0.04 K, respectively, for LADAS compared to ADAS estimates. Regionally, the RMSE of LADAS q2m and T2m_max_ is improved by up to 0.4 g/kg and 0.3 K, respectively. Improvement in LADAS specific humidity extends into the lower troposphere (below ~700 mb), with relative improvements in bias of 15–25%, although LADAS air temperature bias slightly increases relative to that of ADAS. Finally, the root mean square of the LADAS Tb observation-minus-forecast residuals is smaller by up to ~0.1 K than in a land-only assimilation system, corroborating the positive impact of the Tb analysis on the modeled land–atmosphere coupling.

## I. Introduction

SOIL moisture plays an important role in the Earth’s energy, water, and carbon cycles through its control on photosynthesis and evapotranspiration, which in turn impact atmospheric boundary layer dynamics. Consequently, the accurate modeling of soil moisture is critical for improving weather and seasonal climate predictions [1]–[5]. But soil moisture processes and land–atmosphere interactions are highly complex and heterogeneous, and current models are subject to large uncertainties [6]. Errors in modeled land surface fields can be reduced through the assimilation of land surface observations [7]. For example, near-surface air temperature and humidity are sensitive to soil moisture under certain atmospheric conditions. Since the 1990s, many weather centers have been using screen-level (2-m) temperature and humidity measurements to constrain the simulated soil moisture and thereby improve medium-range forecasts of near-surface temperature and precipitation in their operational data assimilation systems [8]–[16].

More recently, satellite observations suitable or specifically designed for the estimation of global surface soil moisture have become available. The sensors and platforms of most relevance for land assimilation include the Advanced Scatterometer (ASCAT, since 2007) [17], [18], the Soil Moisture and Ocean Salinity (SMOS) mission (since 2010) [19], [20], and the Soil Moisture Active Passive (SMAP) mission (since 2015) [21], [22]. ASCAT measures C-band (5.3 GHz) radar backscatter, which is sensitive to moisture in the top ~1 cm soil layer, whereas SMOS and SMAP collect L-band (1.4 GHz) passive microwave brightness temperature (Tb) observations, which are highly sensitive to moisture in the top ~5 cm soil layer. Soil moisture retrievals from these sensors are now assimilated in the operational data assimilation systems at several weather centers, with a positive or neutral impact on short- and medium-range forecasts of screen-level temperature and humidity [12], [16], [23]–[26].

SMAP observations have been used operationally since 2015 in the NASA Goddard Earth Observing System (GEOS) land data assimilation system (LDAS) to generate the SMAP Level-4 Soil Moisture (L4_SM) product [27]. Using ensemble-based techniques, the L4_SM algorithm optimally combines the information from SMAP Tb observations, the land surface model, and its surface meteorological forcing data. The resulting L4_SM soil moisture estimates have the advantage of complete coverage in space and time, including the propagation of surface layer information from SMAP observations into deeper soil layers, and were shown to improve upon model-only soil moisture estimates [28], [29].

The L4_SM algorithm is a stand-alone, land-only data assimilation system forced with surface meteorological data from the quasi-operational, near-real-time GEOS Forward Processing (FP) atmospheric data assimilation system (ADAS) [30]. The L4_SM information does not, however, feed back into the FP ADAS, which does not yet include a land surface analysis. Likewise, the GEOS ADAS version that generates the Modern-Era Retrospective Analysis for Research and Applications, version 2 (MERRA-2) [31] product does not include a land surface analysis, although the MERRA-2 system uses gauge-based precipitation to force the land surface, which mitigates some of the errors in the modeled soil moisture [32].

To explore the potential benefit of assimilating land surface observations in future NASA reanalyses, Draper and Reichle [33] (hereinafter DR19) developed a weakly coupled GEOS land–atmosphere data assimilation system (LADAS). In this weakly coupled framework, the atmospheric analysis and the land surface analysis are performed separately, without sharing observation or model background error information. The two-way interactions of the LDAS and ADAS subsystems in the LADAS are predicated on the 6-h ADAS assimilation cycle and consist of 1) forcing the LDAS with surface meteorological data from the ADAS and 2) forcing the ADAS with soil moisture correction terms generated by the LDAS (see Section II). With the ADAS subsystem of their LADAS configured to match that of MERRA-2, DR19 showed that the assimilation of soil moisture retrievals from ASCAT and SMOS improved estimates of screen-level temperature and humidity in the LADAS when compared to the control ADAS experiment without a land surface analysis.

SMAP can provide more accurate and more spatially complete soil moisture information than ASCAT or SMOS [34]–[36]. The primary objective of this article is to determine by how much the assimilation of SMAP Tb observations in the GEOS LADAS can improve the simulated soil moisture and near-surface atmospheric variables. The design of the LADAS and the overall experimentation approach are as in DR19. However, this study differs from DR19 in three key aspects. First, the LDAS used here employs a spatially distributed (“three dimensional”) land analysis and directly assimilates SMAP Tb observations (that is, radiances) after a seasonally varying Tb bias correction as in the L4_SM algorithm (see Section II-B), whereas DR19 used a local (“one dimensional”) assimilation of ASCAT and SMOS soil moisture retrievals after cumulative distribution function matching [37]. Both radiance and retrieval assimilation are commonly used, although the two approaches differ in how the observations add value to the system [38]. Second, compared to DR19, the LADAS used here includes improved atmospheric and land models [28], [39], and the atmospheric analysis in the ADAS now assimilates an expanded suite of atmospheric observations (see Section II-B). Third, unlike DR19, the present study does not use gauge-based precipitation to force the land surface because the present study focuses on the near-real-time GEOS FP weather analysis, for which the gauge-based precipitation data do not meet latency requirements. The latter two differences from DR19 have a competing impact on the ADAS baseline skill against which improvements from SMAP data assimilation are measured.

As will be demonstrated in this study, SMAP assimilation in the LADAS yields improved estimates of soil moisture and near-surface atmospheric variables compared to the ADAS baseline, and the skill improvements from SMAP assimilation are slightly better than those documented by DR19 for ASCAT and SMOS assimilation. An enhanced GEOS land-atmosphere analysis has the potential to improve a range of GEOS data products, including the near-real-time GEOS FP weather analysis and prediction products, the GEOS subseasonal-to-seasonal forecasts, and future GEOS-based reanalysis products.

The rest of this article is organized as follows. Section II describes the data and methods that are used in the data assimilation experiments and for the evaluation. Section III assesses the results of the assimilation experiments. Finally, Section IV provides a summary and plans for future development.

## II. Data and Methods

### A. SMAP Observations

Since 31 March 2015, the SMAP observatory has been measuring L-band (1.4 GHz) passive microwave Tb with full global coverage every 2–3 days. The SMAP Tb observations are highly sensitive to surface soil moisture and temperature in regions with less than ~5 kg·m^−2^ vegetation water content. The SMAP Tb observations used here are from the L1C_TB Version 4 product [40] on the 36-km Equal-Area Scalable Earth version 2 (EASEv2) grid [41]. Prior to assimilation, we average the fore- and aft-looking Tb data. We assimilate horizontally (H) and vertically (V) polarized Tb observations from descending and ascending half-orbits (~6am/pm local equator overpass time, respectively).

The total Tb observation error standard deviation is prescribed to a constant value of 4 K, which includes the instrument error and the representativeness error associated with the forward radiative transfer model that converts the simulated soil moisture and temperature into model Tb estimates (see Section II-B). The observation errors of H- and V-polarization Tb are assumed to be uncorrelated. The Tb observations are screened in a quality control procedure before they are accepted into the assimilation. For instance, we exclude observations that are flagged for nonoptimal quality in the L1C_TB product or fall outside the natural range (100–320 K). Moreover, we exclude observations for times and locations with frozen or snow-covered surface conditions (based on soil temperature and snow mass estimates in the LDAS) when the radiative transfer model is not valid [28]. On average, during the June–August (JJA) 2017 experimental period, approximately one pair of H- and V-polarization observations from each 36-km land grid cell in the L1C_TB product was assimilated every day in the midlatitudes, with higher counts in the high latitudes and lower counts in the Tropics owing to SMAP’s orbital characteristics.

### B. Data Assimilation Systems

#### 1) Atmospheric Data Assimilation:

**Fig. 1.**
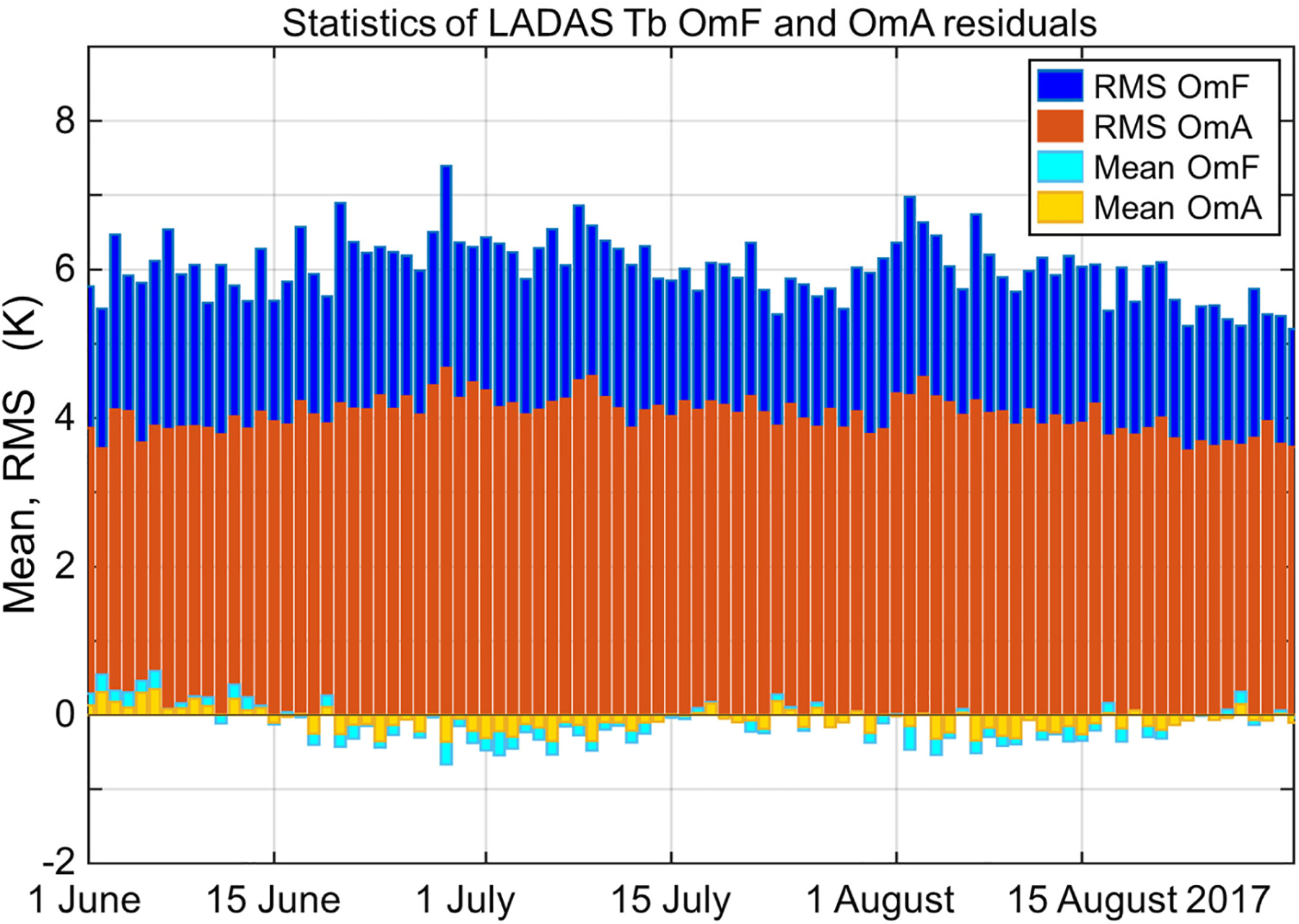
Daily global mean and RMS of L-band Tb OmF and OmA residuals from the LDAS subsystem in the LADAS experiment for JJA 2017. OmF and OmA residuals computed after climatological rescaling of SMAP Tb observations.

In this study, we used the GEOS ADAS version 5.26.4. As in MERRA-2 and DR19, the AGCM was set up on a 0.5° resolution (“c180”) cubed-sphere horizontal grid with 72 hybrid-eta levels from the surface to 0.01 hPa, with a lowest atmospheric model layer centered at ~40–60 m above the surface. Model output was written on a 0.5° by 0.625° latitude–longitude grid. The latter grid was also used for the atmospheric data assimilation, which used a three-dimensional variational deterministic (3D-Var) analysis. Each 6-h assimilation cycle starts with an atmospheric analysis that merges observations with a model background from the previous cycle’s AGCM “first guess” background forecast (or “Predictor” segment) and thereby produces atmospheric correction terms (or “increments”). Next, another AGCM simulation (or “Corrector” segment) for the same 6-h period is forced with tendency terms derived from the atmospheric analysis increments. Finally, the next Predictor segment (i.e., an AGCM forecast for the next 6-h period) is generated to complete the cycle. See [42, Fig. 1] for a schematic.

The ADAS resolution and the 3D-Var atmospheric analysis method used here match that of MERRA-2 and DR19. The ADAS version 5.26.4 used in this study, however, includes several important upgrades from that of MERRA-2 and DR19 (ADAS version 5.12.4). Specifically, the AGCM used here includes important upgrades in the land physics [28], [43], the atmospheric physics and radiation [39], [44], [45], and the atmosphere–ocean interface parameterization [46]. Furthermore, the atmospheric analysis now benefits from an expanded set of assimilated atmospheric observations taken here from the GEOS operational data stream as in FP (most notably, now including data from the Special Sensor Microwave Imager Sounder), an ocean skin temperature analysis [46], retuned background error covariances, the use of interchannel observation error correlations for handling data from the Atmospheric Infrared Sounder and the Infrared Atmospheric Sounding Interferometer [47], and the application of a digital filter to the atmospheric analysis tendencies in the incremental analysis update procedure [48], [49]. Finally, unlike MERRA-2 and DR19, this study does not use gauge-based precipitation data to correct the land surface forcing in the ADAS (to be consistent with FP latency requirements).

#### 2) Land Data Assimilation:

The GEOS LDAS consists of a stochastic ensemble Kalman filter (EnKF) [38], [50], [51] and the Catchment land surface model [52], [53]. Unlike traditional, layer-based land surface models, the Catchment model explicitly accounts for spatial variations in soil moisture and water table depth within each “tile” (or computational unit) of a watershed based on its topographic statistics. Specifically, the Catchment model simulates soil moisture in three nested layers for the surface (0–5 cm), root-zone (0–100 cm), and profile (0 cm to bedrock), based on the spatially varying equilibrium soil moisture profile (where gravity balances capillary forces) and deviations thereof in the surface and root-zone layers. The model prognostic variables accounting for these deviations are the “surface excess” and “root-zone excess,” respectively. A third model prognostic variable, the “catchment deficit,” tracks the amount of water that would be needed to bring the catchment to full saturation. Volumetric soil moisture estimates in the three nested layers are diagnosed from these three model prognostic variables. The model also includes a six-layer heat diffusion model for soil temperature and a three-layer snow model component that describes the state of the snowpack in terms of snow water equivalent, snow depth, and snow heat content [54].

The core of the observation operator is a zero-order “tau-omega” radiative transfer model [55] that converts the simulated soil moisture and temperature into model estimates of the observed L-band Tb. Key input parameters to the radiative transfer model, such as the microwave surface roughness, vegetation structure parameters, and scattering albedo, were calibrated using multiangular SMOS Tb observations [28], [56].

The LDAS used here matches that of the Version 4 L4_SM algorithm [28], except that here the Catchment model is set up in the 1/2-degree cube-sphere tile space, uses look-up table values for tree height to compute surface aerodynamic roughness lengths, and employs the Helfand surface turbulence scheme [28], [43]. These modifications are necessary to make the Catchment model version and configuration in the LDAS fully consistent with that in the AGCM of the ADAS. The Catchment model is driven with surface meteorological forcing data from the ADAS. Ensemble spread in the land states is achieved by applying random perturbations to select surface meteorological forcing data and land model prognostic variables as described in [27]. Every three hours, the available SMAP Tb observations and corresponding model forecasts are used in an EnKF analysis, which computes increments for the surface excess, root-zone excess, surface temperature, and surface soil heat content of each ensemble member. These increments are then immediately applied to correct the soil moisture and temperature state in the respective Catchment model ensemble members.

Seasonally-varying bias in the modeled Tb is addressed prior to data assimilation by converting the Tb observations and the model-simulated Tb into anomalies from their respective mean seasonal cycles. This is done separately for each grid cell, polarization, and orbit direction [38]. Here, the required mean seasonal cycles were estimated from SMAP observations available for the period from April 2015 to June 2020. The mean seasonal cycles for the model-simulated Tb were sampled at the times and locations of the SMAP overpasses from an “Open Loop” ensemble simulation of the land modeling system (that is, with the application of perturbations but without SMAP Tb assimilation; Section II-C). This approach ensures a largely unbiased land surface analysis, at the expense of not correcting potential errors in the model’s mean seasonal cycle.

#### 3) Coupled Land and Atmospheric Data Assimilation:

The GEOS LADAS used here is built on the recent GEOS ADAS and LDAS versions described above but otherwise follows the basic structure described in DR19; their Fig. 1 provides a schematic diagram. Building on the 6-h Predictor/Corrector cycle of the ADAS described above, the ADAS and LDAS subsystems are weakly coupled through the land–atmosphere interactions encoded in the AGCM, which provides surface meteorological forcing data to and receives land analysis increments from the LDAS subsystem. Specifically, in parallel to the atmospheric analysis, surface meteorological forcing data from the previous cycle’s AGCM background forecast (i.e., Predictor segment) are used to drive a Catchment model ensemble simulation in the LDAS for the current 6-h analysis period. As part of this simulation, the LDAS assimilates the available SMAP observations and produces land analysis increments in two 3-h land analysis steps. The AGCM simulation during the Corrector segment is then forced with these land increments as well as the atmospheric tendency terms derived from the atmospheric analysis.

### C. Experimental Design

A suite of three data assimilation experiments, summarized in Table I, was carried out to determine the impact of SMAP Tb assimilation on the skill of the simulated soil moisture and near-surface atmospheric variables. The control experiment (ADAS) assimilates the full stream of atmospheric observations as in FP operations, albeit in the coarser-resolution, 3D-Var ADAS configuration described above (see Section II-B). The ADAS experiment does not assimilate SMAP Tb. The second experiment (LADAS) is like the ADAS control experiment but with the additional assimilation of SMAP Tb observations in the weakly coupled LADAS configuration (Section II-B). The LADAS experiment includes two-way feedback between the assimilation of SMAP Tb observations at the land surface and the atmospheric model and analysis. (Note that the ADAS control experiment is identical to running the LADAS without SMAP Tb assimilation because the only impact from the LDAS on the ADAS subsystem within the LADAS is through the soil moisture and temperature increments generated in the SMAP Tb analysis.) Finally, for reference, we also conducted a land-only (“offline”) assimilation experiment (LDAS_offl_) that is forced with surface meteorological data from the Predictor segment of the ADAS control experiment and assimilates only SMAP Tb observations without feeding back on the atmospheric model or analysis.

The experimental period for the three data assimilation experiments is JJA 2017. To allow for spin up, the AGCM in the ADAS control experiment was initialized on 1 April 2017 at 2100 UTC from MERRA-2 except for the land surface, which was initialized from a separate Catchment model simulation to address the structural land model changes between MERRA-2 and the recent GEOS version used here (Section II-B). The latter, land-only, multidecadal spin-up simulation consisted of a single member without perturbations and was driven with surface meteorological forcing data from MERRA-2 through 2014 and from FP thereafter. The AGCM in the LADAS experiment was initialized from that of the ADAS control experiment on 3 May 2017 at 2100 UTC, allowing for a four-week spin-up of the land analysis feedback. The 24-member land ensemble of the LDAS subsystem in the LADAS experiment was initialized from the LDAS ensemble Open Loop simulation (see Section II-B), which was in turn initialized on 1 January 2015 at 0000 UTC from the above-mentioned land-only spin-up simulation. Finally, the LDAS_offl_ experiment was initialized on 31 May 2017 at 2100 UTC from the LDAS ensemble in the LADAS experiment (see Table I).

### D. Data and Methods for Evaluation

This section describes the metrics and independent *in situ* measurements used to evaluate the output from the assimilation experiments. Depending on the variable, the performance metrics used here include the bias, unbiased root-mean-square error (ubRMSE, or standard deviation of the error) [57] and correlation (R) versus the *in situ* measurements. We also estimate 95% confidence intervals for soil moisture correlation and ubRMSE following [28]. Estimates of soil moisture bias computed from point-scale *in situ* measurements are dominated by the large and unavoidable systematic errors in spatial upscaling, even for the locally dense SMAP core validation sites described below [58]. Hence, statistical confidence intervals are not shown for soil moisture bias estimates, and the differences in soil moisture bias estimates seen below are not considered statistically significant.

The *in situ* soil moisture measurements used here fall into two categories. 1) SMAP core validation sites with locally dense sensor networks that provide *in situ* measurements at the scale of the satellite measurements and model estimates [59]–[74]; and 2) the so-called sparse networks with typically just one or two point-scale sensor profiles per satellite footprint, including the USDA Natural Resources Conservation Service Soil Climate Analysis Network [75], the U.S. Climate Reference Network [76], [77], the Oklahoma Mesonet [78], the SMOSMANIA network in the southwest of France [79], and the OzNet network in Australia [80].

Here, we use upscaled (33-km) surface (root-zone) soil moisture measurements for 12 (6) core sites. The sites match those in [81, Tab. 1], except here we do *not* use surface measurements from Reynolds Creek, Ngari, St Josephs, Tonzi Ranch, Niger, and Benin or root-zone measurements from Yanco for lack of sufficient *in situ* data during the JJA 2017 experimental period. Processing of the core site measurements follows [27]. The simulated soil moisture values are mapped to the core site locations using nearest-neighbor interpolation. Surface (root-zone) soil moisture measurements were available and used here from 360 (228) sparse network sites, which were grouped into 108 (76) clusters for the purpose of computing average metrics (for details, [81, see Section 6.3 and Table 2]). The *in situ* soil moisture measurements provide invaluable information but are subject to measurement errors and offer very limited coverage outside of the contiguous United States.

Following DR19, the simulated screen-level (2-m) specific humidity (q2m) and air temperature (T2m) are evaluated versus *in situ* measurements collated by the Hadley Centre Integrated Surface Database (HadISD version 3.1.1.202007p) [82], [83] and the Global Historical Climatology Network (GHCN; GHCN-DAILY version 3.26) [84], respectively. HadISD provides subdaily (between hourly and six hourly), station-based, quality-controlled q2m data from ~7000 stations during JJA 2017. GHCN provides station-based, quality-controlled measurements of daily maximum T2m (T2m_max_) from ~12000 stations. The stations in HadISD and GHCN are unevenly distributed across the globe, with good coverage in much of North America and Eurasia but generally poor coverage in the high latitudes, South America, Africa, the Tibetan Plateau, and central Australia (not shown). The AGCM-simulated q2m and T2m are diagnosed (interpolated) from the corresponding values at the surface and in the lowest atmospheric model layer using stability functions. The simulated q2m is written out at hourly intervals, mapped to each station location using a nearest-neighbor approach, and compared to the HadISD q2m whenever an observation is available. For each day and model grid cell, the simulated T2m_max_ is determined from hourly AGCM output and mapped from the model grid to the GHCN station location using a nearest-neighbor approach. To improve the clarity of the illustrations, the metrics from the individual stations are averaged and plotted on a 2° latitude–longitude grid.

The 6-h AGCM forecasts that provide the model background state during each assimilation cycle (Section II-B; see Fig. 1 of DR19 for a schematic diagram) contain crucial information about the cumulative impact of the observations that were assimilated in all preceding cycles. Ideally, these forecasts match—within the assumed (that is, prescribed) model and observation error statistics—the corresponding observations, which have not yet been assimilated. The forecasts should be unbiased, and more accurate forecasts generally indicate a higher quality analysis. We, therefore, examine key statistics of the observation-minus-forecast (OmF) residuals for the simulated L-band land surface Tb and atmospheric profiles of air temperature and humidity, including their mean, root mean square (RMS), and standard deviation. The OmF statistics for the L-band Tb are computed using SMAP Tb observations (after the climatological adjustment applied prior to assimilation; Section II-B). The OmF statistics for air temperature and humidity profiles are computed against 6-h radiosonde observations at six vertical levels between 1000 and 300 mb from 397 locations over continental land, which we obtained from the NOAA Meteorological Assimilation Data Ingest System.^1^

## III. Results and Discussion

### A. Tb Residuals and Soil Moisture Increments

The LDAS subsystem in the LADAS is designed to minimize the disagreement between the SMAP-observed and model-simulated Tb. The proper functioning of the LDAS in its land-only configuration was thoroughly verified during repeated validation of the L4_SM product [27]–[29], [51]. Since here the LDAS is used within the coupled LADAS, at a different resolution, and in the Catchment model’s cube-sphere tile space (Section II-B), this section confirms the proper functioning of the LDAS subsystem by briefly examining the Tb residuals and the resulting soil moisture analysis increments.

Fig. 1 shows the daily global mean and RMS of the Tb OmF residuals from the LADAS, along with the same for the Tb observation-minus-analysis (OmA) residuals. The OmF residuals provide independent verification of the model forecast Tb (see Section II-D). In contrast, OmA residuals are computed by differencing a SMAP Tb observation and a model forecast that was informed by this very observation. The daily variations in the OmF and OmA statistics are primarily caused by seasonal and weather-driven changes in land surface conditions. The daily coverage changes stemming from SMAP’s eight-day exact repeat orbit play only a minor role. Across the experimental period, the daily mean Tb OmF and OmA values (cyan and orange bars, respectively) typically range from −0.5 to 0.5 K, with time-series averages of −0.11 K for the OmF and −0.08 K for the OmA residuals (see Fig. 1). The small magnitude of the mean Tb OmF residuals confirms that the Tb analysis is largely free of bias, which is primarily a consequence of the climatological adjustment of the SMAP Tb observations prior to their assimilation (see Section II-B).

The daily global RMS of the Tb OmF residuals (blue bars in Fig. 1) ranges from 5 to 7.5 K, with a time series average (computed in quadrature) of 6.0 K. These values measure the typical misfit between a SMAP Tb observation and the corresponding model forecast for a given time and location. Finally, the daily RMS of the Tb OmA residuals (red bars) ranges from 3.5 to 4.5 K, with a time series average of 4.1 K; the reduction by ~2 K from the Tb OmF RMS is the result of the Tb analysis, which by design brings the model estimate closer to the assimilated observation. The OmF and OmA statistics seen here are fully consistent with those of the SMAP L4_SM algorithm [28]. This finding provides confidence in the proper workings of the LDAS subsystem in the cube-sphere tile space employed here (as opposed to the 9-km EASEv2 grid setup of the L4_SM algorithm).

**Fig. 2.**
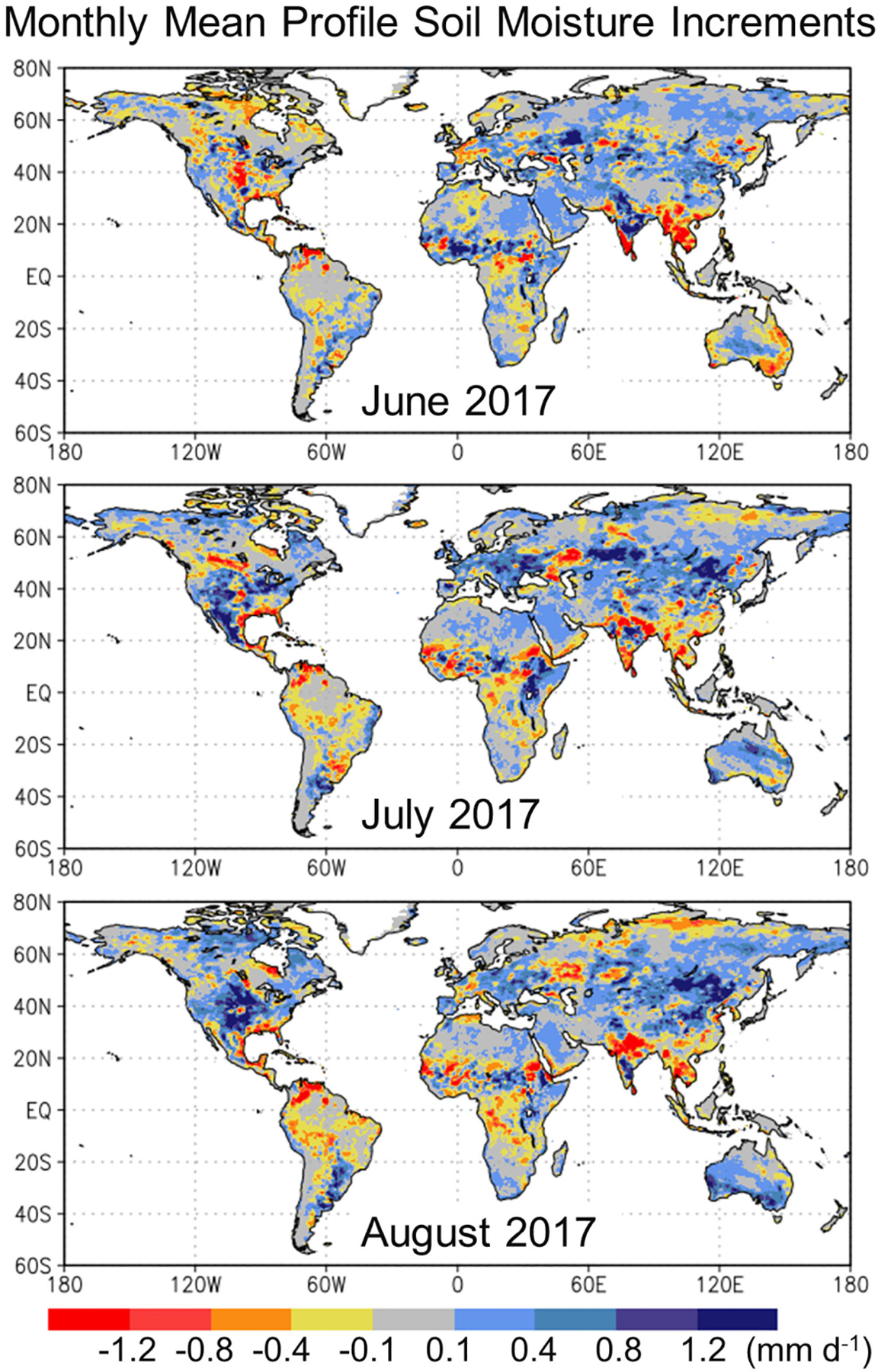
Monthly mean of ensemble-average profile soil moisture increments in equivalent flux units (mm·d^−1^) in the LADAS experiment for (a) June, (b) July, and (c) August 2017.

Next, Fig. 2 illustrates the monthly mean of the analysis increments for the total profile soil moisture in equivalent flux units (mm d^−1^), separately for June, July, and August 2017. The 3-h soil moisture increments underpinning the graphic were computed by the LDAS subsystem and then applied to the AGCM’s land surface states in the ADAS subsystem of the LADAS. Owing to the nearly bias-free Tb analysis (see Fig. 1), the soil moisture increments mostly vanish when averaged across longer periods [28]. For individual months, however, the monthly mean soil moisture increments typically range from -1.5 to 1.5 mm·d^−1^. For example, in June 2017, there was a strong negative (drying) increment in the center of the continental U.S. [see Fig. 2(a)], whereas in August 2017, the same region experienced a strong positive (wetting) increment [see Fig. 2(c)]. Compared to the monthly mean soil moisture increments of DR19 (their Fig. 5) for summer 2013, the increments seen here are more variable in space and time. This is primarily a consequence of the different bias correction strategies. DR19 assimilated soil moisture retrievals after cumulative distribution function matching [37]. Their approach does not remove seasonally-varying bias, which, if present, can dominate the signal in the soil moisture increments. Here, this mean seasonal bias is removed and only the anomaly signal in the SMAP Tb observations is assimilated (see Section II-B).

**Fig. 3.**
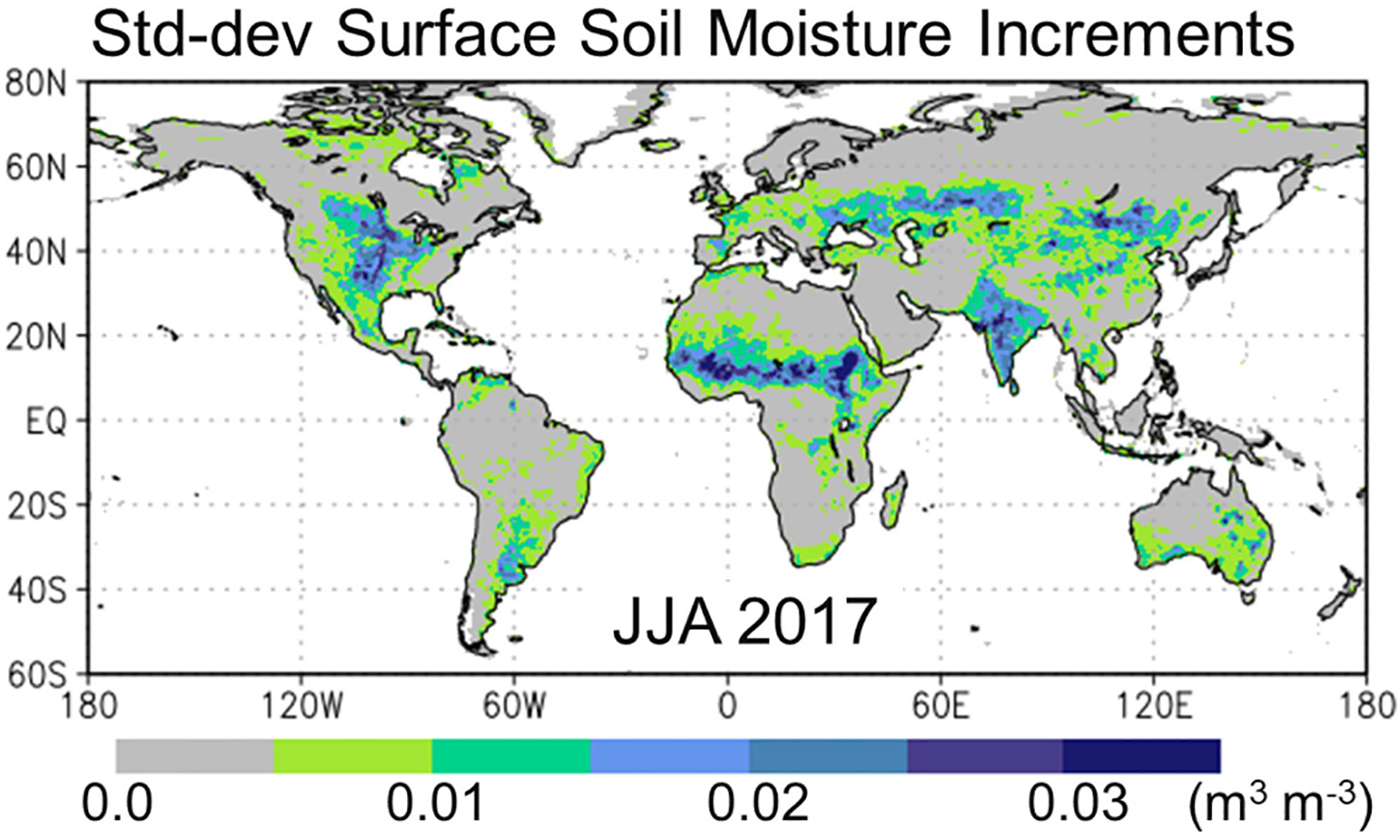
Time series standard deviation of ensemble-average analysis increments for surface soil moisture (m^3^·m^−3^) in the LADAS experiment for JJA 2017.

Finally, Fig. 3 shows the standard deviation of the surface soil moisture analysis increments, which measures the typical 3-h correction in the 0–5 cm surface layer resulting from a SMAP Tb analysis. (The statistic excludes increments that are trivially zero because at the time and location, there was not a SMAP overpass within the ~1.25° radius of influence, or because the SMAP Tb observation did not pass quality control [51].) Regions with larger typical increments tend to be in the transition zones between wet and dry climates, including central North America, the Sahel, Central Eurasia, and India (see Fig. 3). These regions also coincide with croplands (e.g., [85, Fig. A2.2]), where agricultural practices such as tilling, harvesting, or irrigation impact the SMAP Tb observations but are not represented in the LDAS modeling system, which can result in large Tb OmF values and, ultimately, large soil moisture increments (even though some systematic errors are removed through the climatological rescaling of the Tb observations prior to their assimilation into the land surface model; Section II-B). Like the Tb OmF statistics, the spatial pattern and typical magnitudes of the increments standard deviation are consistent with those seen in the L4_SM algorithm [28, Fig. 7(c)]. But the pattern is different from that seen for profile soil moisture increments in DR19 [33, Fig. 5], which were relatively large throughout the high latitudes. The likely reason for the difference in the patterns is the aforementioned difference in the bias correction strategy applied prior to assimilation in DR19’s retrieval assimilation.

### B. Evaluation Using In Situ Measurements

**Fig. 4.**
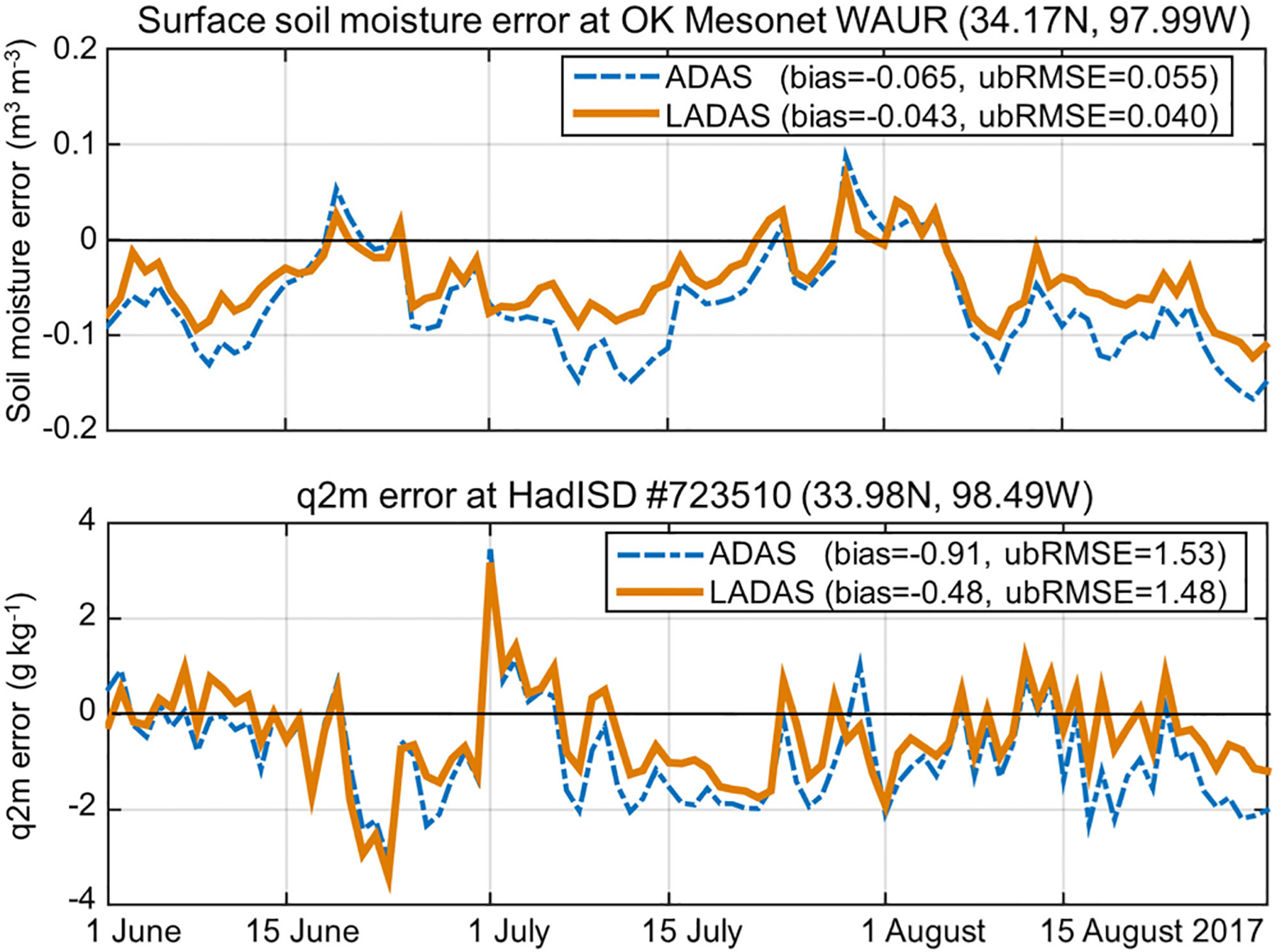
Daily averaged analysis error (model minus measurement) for (a) surface soil moisture (m^3^·m^−3^) and (b) q2m (g·kg^−1^) from the (dashed blue lines) ADAS control and (solid brown lines) LADAS experiments near 34 N, 98 W in Oklahoma U.S. for JJA 2017. Error computed versus *in situ* measurements from the Oklahoma Mesonet Waurika station for soil moisture and from HadISD station #723510 for q2m.

Fig. 4 illustrates, for a representative location, the impact of the SMAP Tb assimilation on the quality of the simulated soil moisture and q2m in the LADAS experiment. During the JJA 2017 experimental period, the error in the ADAS-simulated (control) surface soil moisture versus *in situ* measurements from the Waurika (WAUR) station in the Oklahoma Mesonet ranges from −0.15 to 0.1 m^3^·m^−3^, with a bias of −0.065 m^3^·m^−3^ and an ubRMSE of 0.055 m^3^·m^−3^ [see Fig. 4(a)]. (Note that the point-scale measurements provided by the station are not necessarily representative of the simulated grid-cell scale time average conditions.) The SMAP Tb assimilation in the LADAS reduces the surface soil moisture error by ~30%, resulting in a bias of −0.043 m^3^·m^−3^ and an ubRMSE of 0.040 m^3^·m^−3^ for the LADAS. The improvements extend to the simulated q2m [see Fig. 4(b)]; at a nearby HadISD station (#723510), the q2m bias is reduced from −0.91 g·kg^−1^ in the ADAS control to − 0.48 g·kg^−1^ in the LADAS, and the q2m ubRMSE is slightly reduced from 1.53 g·kg^−1^ in the ADAS control to 1.48 g·kg^−1^ in the LADAS. There is good temporal consistency in the soil moisture and q2m improvements; errors in both variables are most reduced during the first half of July and in the second half of August.

#### 1) Evaluation of Soil Moisture:

**Fig. 5.**
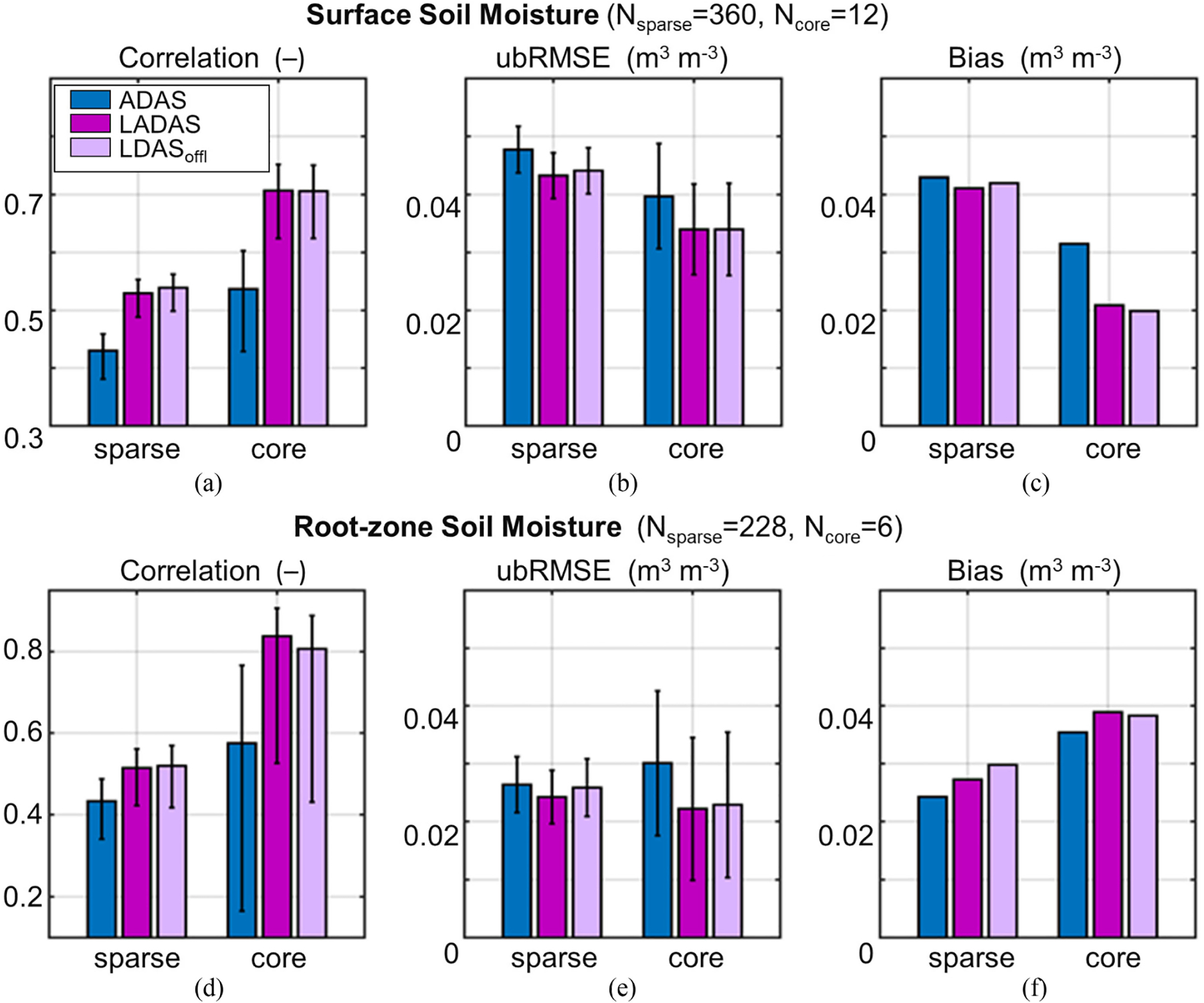
(a), (d) Correlation (dimensionless), (b), (e) ubRMSE (m^3^·m^−3^), and (c), (f) bias (model minus measurement; m^3^·m^−3^) for (a)–(c) surface and (d)–(f) root-zone soil moisture from the ADAS control, LADAS, and LDAS_offl_ experiments. Metrics are computed versus *in situ* measurements from sparse networks (first group of bars) and SMAP core validation sites (second group of bars) for JJA 2017. *N*_sparse_ and *N*_core_ indicate the number of sites used to compute the metrics. Error bars indicate 95% confidence intervals (see text for details). Note that the sparse network and core sites differ in their coverage across land surface conditions and climate zones.

The errors shown in Fig. 4 are just representative examples for a single location. Next, we examine the correlation, ubRMSE, and bias of the simulated, 3-h soil moisture when averaged across the sparse network sites and (separately) the core validation sites (see Fig. 5). LADAS correlation skills for surface and root-zone soil moisture are ~0.5 at the sparse network sites and ~0.7–0.8 at the core sites [see Fig. 5(a),(d)]. At the sparse network sites, the correlation skill of surface and root-zone soil moisture in the LADAS exceeds that of the ADAS control by ~0.1. The skill increase is even larger at the core sites, with the LADAS correlation exceeding that of the ADAS control by 0.17 for surface and 0.26 for root-zone soil moisture. Because the estimated 95% confidence intervals do not overlap, the surface soil moisture skill improvements in the LADAS can be considered statistically significant, despite the relatively small sample size (owing to the short record and few stations available for validation).

The ubRMSE of the LADAS surface (root-zone) soil moisture is 0.043 (0.024) m^3^·m^−3^ at the sparse network sites and 0.034 (0.022) m^3^·m^−3^ at the core sites [see Fig. 5(b),(e)]. The LADAS ubRMSE values are all smaller than those of the ADAS control. Even though the ubRMSE reductions are small (0.002–0.008 m^3^ m^−3^) and not statistically significant, they are consistent across the surface and root-zone soil moisture and for the sparse network and core validation sites. The LADAS soil moisture is generally wetter than indicated by the *in situ* measurements, with an average bias in the surface (root-zone) soil moisture of 0.041 (0.027) m^3^·m^−3^ at the sparse network sites and 0.021 (0.039) m^3^·m^−3^ at the core sites [see Fig. 5(c),(f)]. Compared to the ADAS control, the LADAS bias is improved for surface soil moisture but degraded for root-zone soil moisture, consistently for sparse network and core validation sites. The generally larger soil moisture bias seen here (compared to that of the L4_SM product) is mainly because the land model precipitation forcing here is not corrected with gauge-based data. For root-zone soil moisture, the bias even exceeds the random error component (i.e., the ubRMSE).

#### 2) Evaluation of Screen-Level Humidity and Temperature:

Recall that the only difference between the ADAS subsystem within the weakly coupled LADAS and the stand-alone ADAS is the additional forcing of the AGCM during the Corrector segment with the soil moisture increments generated by the SMAP Tb analysis in the LDAS subsystem (see Section II-B). That is, the SMAP Tb analysis impacts the simulated q2m and T2m primarily through the land–surface interactions encoded in the AGCM; the LDAS does not generate q2m or T2m increments, nor are q2m and T2m measurements assimilated in the ADAS control or LADAS. Consequently, we can use q2m and T2m measurements for an independent assessment of the quality of the simulated q2m and T2m and thereby determine the impact of SMAP Tb assimilation on the simulated screen-level estimates.

**Fig. 6.**
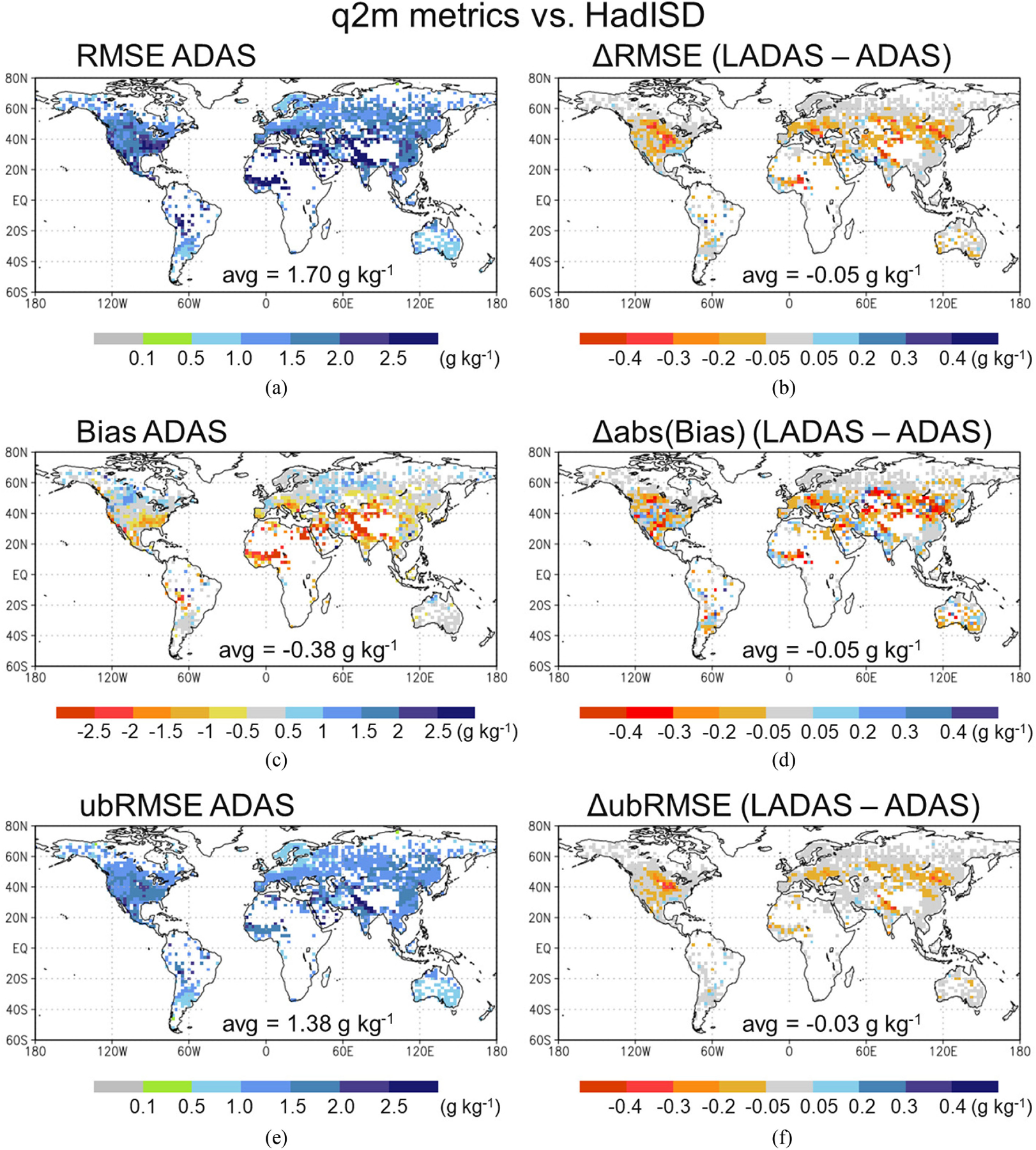
Left column shows (a) RMSE, (c) bias (model minus measurement), and (e) ubRMSE of 2-m specific humidity from the ADAS control experiment versus HadISD measurements. Right column shows LADAS-minus-ADAS difference in the performance statistics for (b) RMSE, (d) absolute bias, and (f) ubRMSE, with red colors in (b), (d), (f) indicating that LADAS has better skill than ADAS. All statistics are calculated for JJA 2017.

Fig. 6 shows the RMSE, bias, and ubRMSE of the ADAS-simulated (control) q2m versus HadISD measurements in the left column and the difference between the LADAS and ADAS control metrics in the right column. (For the bias, the difference is computed after taking the absolute value to determine which bias is smaller in magnitude, independent of the sign of the bias.) The RMSE of the q2m estimates from the ADAS control experiment typically ranges between 1 and 3 g·kg^−1^, with values exceeding 2.5 g·kg^−1^ in some regions, somewhat lower values in the Southern Hemisphere midlatitudes, and a global land average RMSE of 1.70 g·kg^−1^ [see Fig. 6(a)]. The ADAS-simulated q2m is generally too moist by ~0.5–1 g·kg^−1^ in the higher latitudes and too dry by ~1–2 g·kg^−1^ in the Subtropics and Tropics, with a global average dry bias of −0.38 g·kg^−1^ [see Fig. 6(c)]. Consequently, the q2m ubRMSE values are only somewhat smaller than the RMSE values, with a global average of 1.38 g·kg^−1^ [see Fig. 6(e)].

Overall, the assimilation of SMAP Tb in the LADAS improves the q2m estimates compared to those of the ADAS control. The q2m RMSE is considerably improved by ~0.3 g·kg^−1^ in the Northern Great Plains (including Canadian Plains), the western Sahel, and northern India along the Himalayas [see Fig. 6(b)]. Smaller improvements are seen across much of central Eurasia, and only a few small areas show a degradation in the LADAS q2m RMSE, including a slight degradation in the southeastern United States. In the global land average, the q2m RMSE is slightly reduced by 0.05 g·kg^−1^. Much of the q2m RMSE improvement is from a reduction in the (absolute) bias, especially in central Eurasia and Eastern Europe, although some increase in the absolute bias is seen in smaller regions scattered across the globe [see Fig. 6(d)]. In contrast, the q2m ubRMSE shows a smaller improvement across the globe, particularly in the regions where the RMSE is improved the most, including the Northern Great Plains (including Canadian Plains), northern India, and central Eurasia [see Fig. 6(f)]. Like the q2m RMSE, the ubRMSE improvement is very consistent; there is almost no degradation in the LADAS statistic seen anywhere across the globe.

**Fig. 7.**
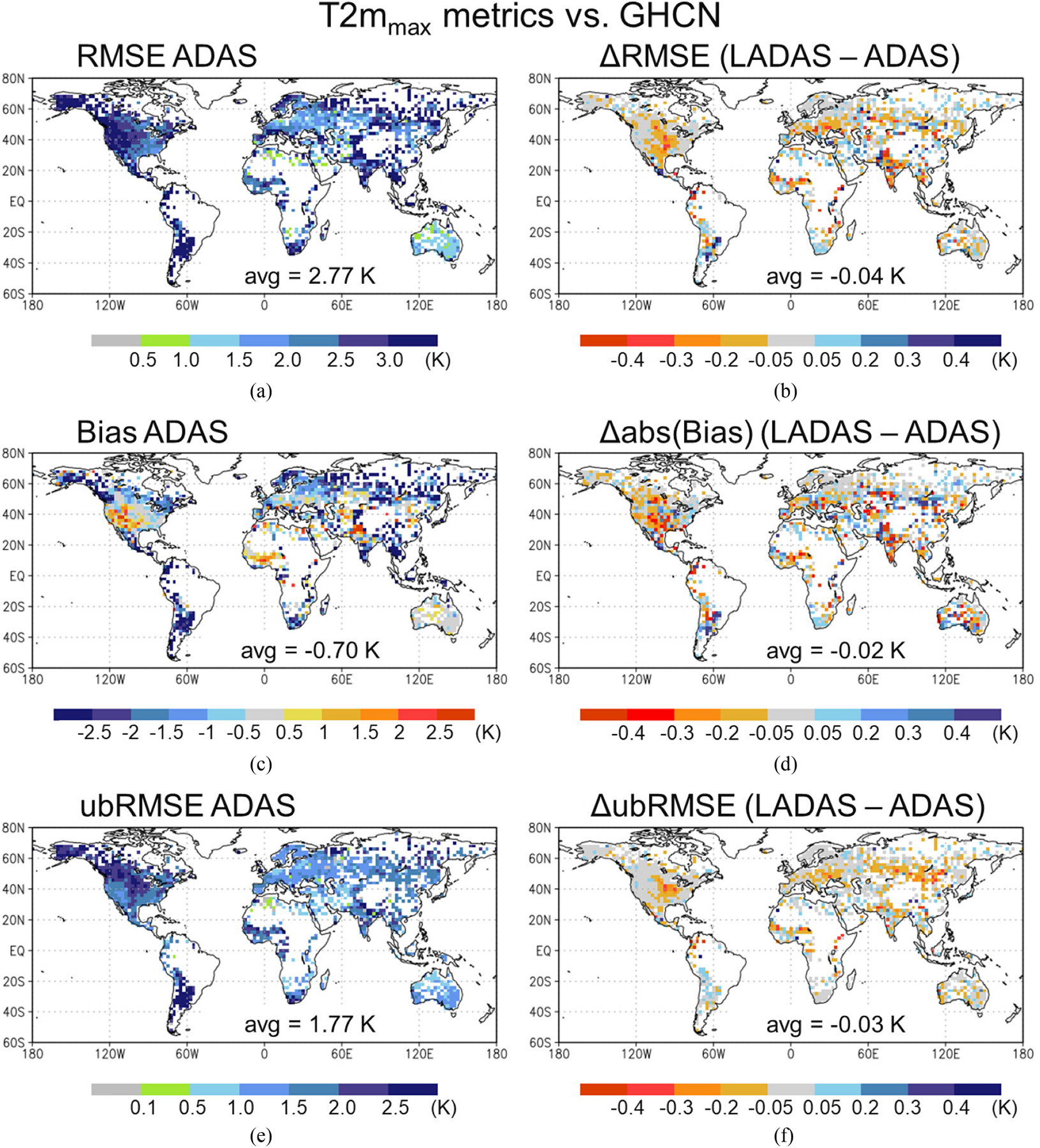
As in Fig. 6 but for 2-m maximum daily air temperature evaluated against GHCN measurements.

Next, Fig. 7 illustrates the impact of the SMAP Tb assimilation on T2m_max_; the figure again shows the ADAS control metrics in the left column and the LADAS minus ADAS differences in the metrics in the right column. The T2m_max_ RMSE for the ADAS ranges from ~1 to 2 K in much of Europe and Australia to ~3.5 K elsewhere, with a global average of 2.77 K [see Fig. 7(a)]. The ADAS T2m_max_ estimates are too cold by 0.7 K on average; they are too cold by ~2 K at the majority of the GHCN stations but too warm 1–2 K in some regions, including the southwestern U.S. and the western Sahel see Fig. 7(b)]. The T2m_max_ ubRMSE is 1.77 K on average and ranges between 0.5 and 1.5 K in much of Europe and Australia and between 1.5 and 3 K in the Americas and Africa [seeFig. 7(c)].

On average, the T2m_max_ RMSE in the LADAS is slightly improved compared to that of the ADAS control, by 0.04 K. The largest RMSE reductions of up to 0.4 K, or ~20% of the RMSE, are seen in the central U.S., the western Sahel, across several subregions of Eurasia, and in central Australia; but there is also some degradation, most notably in Argentina [see Fig. 7(b)]. The net improvement in the (absolute) bias is 0.02 K on average, with a spatial pattern of change like that seen for the RMSE. The ubRMSE in the LADAS is reduced from that of the ADAS control by 0.03 K on average, with improvements of up to ~0.3 K in the above-mentioned regions that showed noteworthy RMSE reductions. The T2m_max_ ubRMSE improvement is also very consistent across the globe; there is only some scattered degradation, typically by less than 0.2 K. Moreover, the improvements in the LADAS T2m_max_ estimates are consistent with those in q2m, as evidenced by their very similar spatial pattern (compare the metrics difference plots of Figs. 6 and 7). This pattern is also consistent with that of the typical magnitude of the soil moisture increments (see Fig. 2).

The q2m and T2m skill metrics (Figs. 6 and 7) seen here are comparable to those of DR19 for mid-April through August of 2013 (their Figs. 9 and 10), with generally consistent spatial patterns. This suggests that the improved modeling system used here at least partly compensates for not using the gauge-based precipitation corrections employed in DR19. Moreover, the improvements in the LADAS q2m and T2m_max_ estimates seen here are also comparable in magnitude to those of DR19 for their ASCAT and SMOS retrieval assimilation. In both studies, the bias improvements contribute more to the RMSE reduction than do the ubRMSE improvements. However, the LADAS improvements seen here include fewer regions of degraded skill and are overall more coherent than those of DR19. Possible reasons for this include the two studies’ differences in the bias correction method applied prior to assimilation, the spatially distributed versus local soil moisture analysis approach, and the generally better soil moisture information provided by the SMAP Tb observations compared to that of the ASCAT and SMOS retrievals.

### C. Impact on Atmospheric Background States

**Fig. 8.**
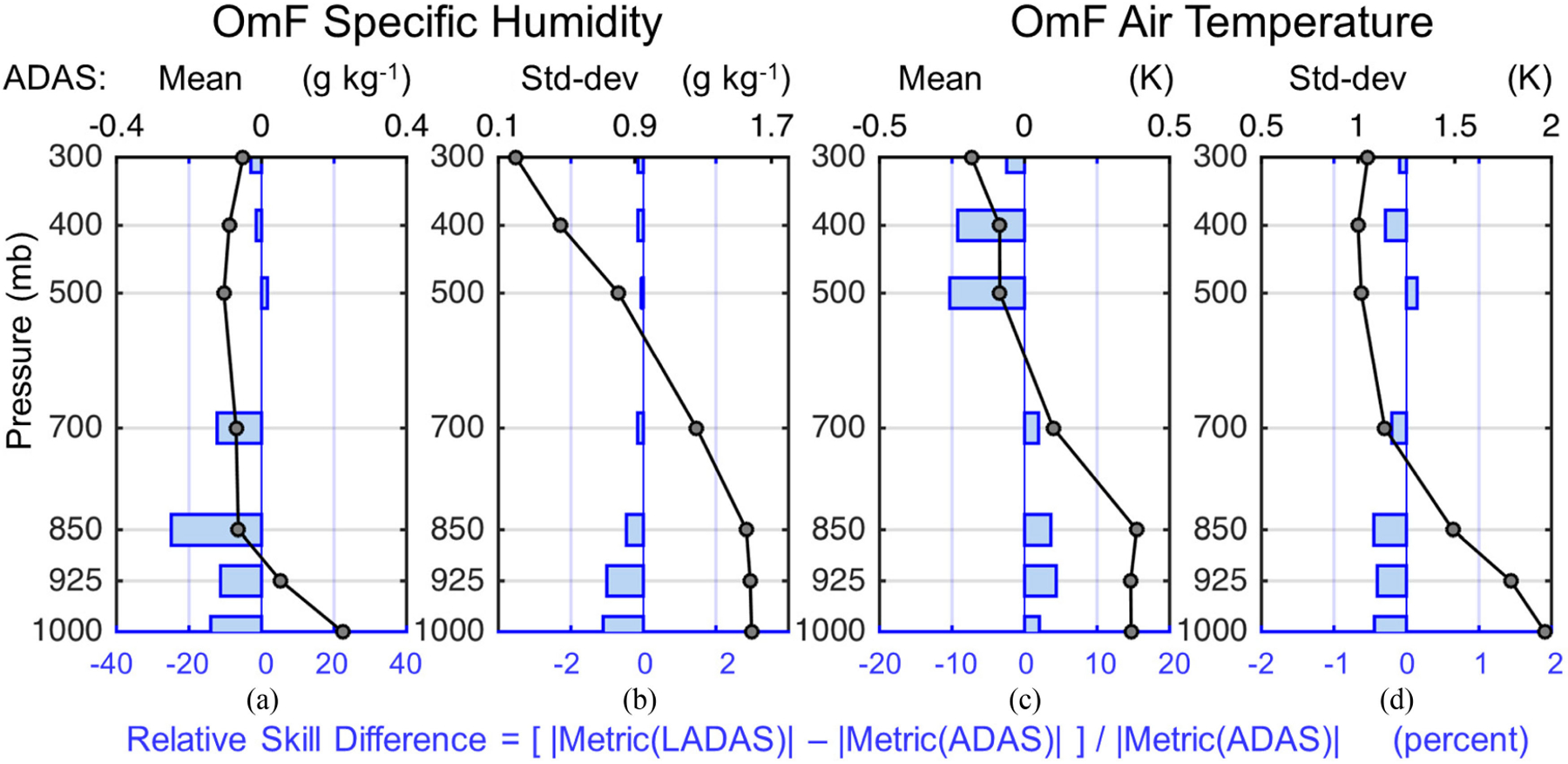
Black dots show atmospheric profiles of the (a), (c) mean and (b), (d) standard-deviation of OmF residuals from the ADAS control experiment for (a), (b) specific humidity (g·kg^−1^) and (c), (d) air temperature (K) across global continental land for JJA 2017. OmF residuals were computed using radiosonde observations. Blue bars indicate corresponding relative skill difference (LADAS versus ADAS) in OmF (a), (c) absolute mean and (b), (d) standard deviation in units of percent. Blue bars with negative (positive) percentage values indicate the better (worse) performance of LADAS compared to ADAS.

In this section, we investigate the impact of the SMAP Tb analysis on the atmospheric background forecast in the ADAS subsystem of the LADAS. The black dots in Fig. 8 show vertical profiles of the JJA 2017 mean and standard deviation of specific humidity and air temperature OmF residuals from the ADAS control experiment, computed using radiosonde observations over continental land only. On average, the ADAS background forecast is too dry by 0.2 g·kg^−1^ at the surface [see Fig. 8(a)], which is consistent with the q2m station comparison of Fig. 6(c). In the lower and middle troposphere, however, the ADAS specific humidity is too moist by 0.1 g·kg^−1^ [see Fig. 8(a)]. There is also a cold bias in the ADAS air temperature of ~0.3 K at the 850–1000 mb levels [see Fig. 8(c)], which is consistent with the screen-level T2m_max_ being too cold on average [see Fig. 7(c)]. The OmF standard deviation for specific humidity is ~1.5 g·kg^−1^ below 500 mb [see Fig. 8(b)], and the OmF standard deviation for air temperature ranges from nearly 2 K close to the surface to ~1 K through the midtroposphere [see Fig. 8(d)].

The blue bars in Fig. 8 indicate the relative skill differences in percentage units between the LADAS and ADAS statistics, with negative values indicating that the (absolute) mean or standard deviation of the LADAS OmF is reduced (improved) from that of the ADAS control experiment and positive values indicating degradation. For specific humidity estimates from the LADAS, the OmF (absolute) mean is improved by 15–25% and the standard deviation is improved slightly by 1–2% in the lower troposphere [see Fig. 8(a),(b)], with a largely neutral impact in the midtroposphere. Near the surface (850–1000 mb), this translates into an improvement of ~0.02–0.03 g·kg^−1^ in the (absolute) mean and an improvement of ~0.01–0.02 g·kg^−1^ in the standard deviation, which is consistent with that seen in the evaluation against screen-level measurements (see Fig. 6). In contrast, air temperature estimates from the LADAS show a small degradation (increase) of 2–3% in the (absolute) bias and a slight improvement of ~0.5% in the standard deviation in the lower troposphere, but both relative differences translate into minimal changes of less than 0.01 K. Similarly, the ~10% improvement in the LADAS air temperature bias from that of the ADAS control at 400–500 mb is minimal (less than 0.01 K) and, given the mixed results in the lower tropospheric temperature, this nominal improvement is most likely noise and not related to the SMAP Tb analysis. In summary, the impact of the SMAP Tb analysis on the atmospheric background forecasts in the LADAS is, as expected, most prominent and beneficial for specific humidity near the surface and mostly neutral in the mid-troposphere.

### D. Weakly Coupled Versus Stand-Alone Land Analysis

**Fig. 9.**
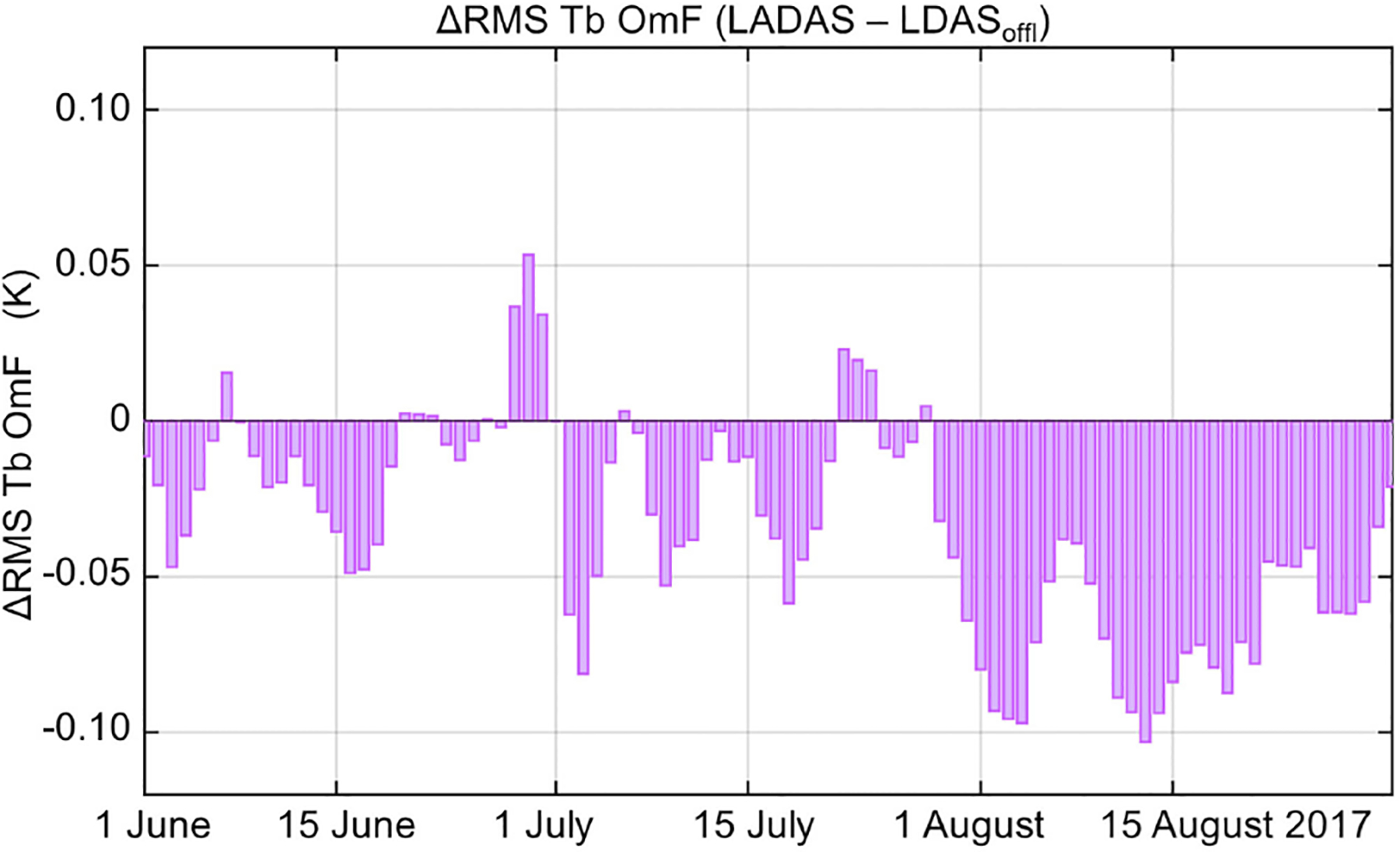
Daily RMS of SMAP Tb OmF residuals from LADAS minus same from LDAS_offl_ for JJA 2017. Negative values indicate that the Tb background forecasts in the LADAS are closer to the SMAP Tb observations than are those of the LDAS_offl_ experiment.

The positive impact of the SMAP Tb analysis on soil moisture, q2m, and T2m_max_ seen above may also improve the quality of the land surface meteorological forcing data in the LADAS, including precipitation, radiation, air temperature, humidity, and wind in the lowest model layer of the AGCM. The improved forcing data may then, in turn, result in improved Tb background forecasts in the LDAS subsystem of the LADAS. Since the latter is forced with data from the AGCM’s Predictor segment in the LADAS, the supplemental, land-only LDAS experiment (LDAS_offl_) was driven with surface meteorological forcing data from the Predictor segment of the ADAS control experiment (Section II-B, Table I). If the SMAP Tb analysis does indeed improve the surface meteorological forcing data in the LADAS, the typical magnitude of the Tb OmF residuals in its LDAS subsystem should be smaller than that of the Tb OmF residuals in the LDAS_offl_ experiment. This expectation is confirmed in Fig. 9, which shows a mostly negative difference in the RMS of the Tb OmF residuals between the LADAS and LDAS_offl_ experiments. The difference is very small during June 2017 but generally increases during the experimental period, which suggests that the LADAS may still be spinning up during much of the JJA experimental period. By August 2017, the typical Tb OmF residuals from the LADAS are smaller by up to ~0.1 K than those from LDAS_offl_. This difference is comparable to the reduction in the Tb OmF RMS achieved with the Catchment model improvements introduced with Version 4 of the L4_SM product [28].

The improvements seen in Fig. 9 indicate that it is beneficial to conduct the SMAP Tb analysis in the weakly coupled system, permitting feedback from the improved soil moisture estimates on the surface meteorological forcing data, which in turn improves the land surface analysis beyond what is possible with “fixed” forcing from an entirely separate ADAS. Further indication of the improvements associated with this coupling is seen in Fig. 5, which includes skill metrics for the LDAS_offl_ experiment in addition to those of the ADAS control and LADAS experiments that were discussed above (see Section III-B). Even though the skill differences between the LADAS and LDAS_offl_ soil moisture estimates are very small and not statistically significant, the surface and root-zone soil moisture ubRMSE for LADAS is slightly but consistently better than that of the LDAS_offl_ experiment for both the core validation and sparse network sites (Fig. 5(b),(e)). Compared to LDAS_offl_, the LADAS also has somewhat better root-zone soil moisture correlation skill at the core sites [see Fig. 5(d)] and lower bias at the sparse network sites [see Fig. 5(c),(f)], although the LADAS has slightly worse correlation at the sparse network sites [see Fig. 5(a),(d)] and slightly worse bias at the core sites [see Fig. 5(c),(f)]. The relatively small magnitude of the differences between the LADAS and LDAS_offl_ metrics is consistent with the multistep nature of the soil moisture feedback on the atmospheric forcing [86].

## IV. Summary and Conclusion

SMAP has been providing L-band microwave brightness temperature observations of unprecedented quality that are highly sensitive to surface soil moisture unless the soil is frozen or obscured by dense vegetation. This study’s main objective is to examine the impact of assimilating SMAP Tb observations in a weakly coupled land–atmosphere data assimilation system. To this end, we use the recently developed GEOS LADAS, which couples the SMAP Tb analysis from the land-only L4_SM algorithm with the GEOS ADAS used for weather analysis. We use the latter in 3D-Var configuration as in MERRA-2, but with the model and atmospheric analysis improvements implemented since MERRA-2. By comparing the LADAS results to those from stand-alone ADAS and LDAS_offl_ experiments, we demonstrate that the SMAP Tb analysis improves the LADAS-simulated soil moisture, screen-level atmospheric variables, and near-surface atmospheric humidity and temperature profiles.

Specifically, we find that the SMAP Tb analysis in the LDAS subsystem of the LADAS yields statistics of the Tb OmF residuals and soil moisture analysis increments that are similar to those generated by the well-tested, land-only L4_SM algorithm (see Figs. 1–3), which confirms the proper functioning of the LDAS subsystem, especially its setup in the cube-sphere tile space used here and its coupling to ADAS subsystem.

Validation versus *in situ* measurements of surface and root-zone soil moisture, q2m, and T2m_max_ demonstrates the beneficial impact of the SMAP Tb analysis in the LADAS. LADAS-simulated surface and root-zone soil moisture have higher correlation and lower ubRMSE than corresponding ADAS control estimates when validated against *in situ* measurements from 12 SMAP core sites and 360 stations across five sparse networks (see Fig. 5). The benefit of the SMAP Tb analysis seen here is fully consistent with prior validation results of the L4_SM product [28], [29]. We further show that LADAS-simulated q2m and T2m_max_ have, on average, slightly lower RMSE, ubRMSE, and (absolute) bias than corresponding ADAS control estimates (see Figs. 6 and 7). There are very few instances of degraded performance, and RMSE values are improved by up to 0.3 g·kg^−1^ for q2m and up to 0.4 K for T2m_max_ in some regions. Improvements are relatively larger in q2m than T2m_max_, which is consistent with the fact that SMAP primarily provides information about the water cycle. The smaller improvements in LADAS screen-level temperature and humidity estimates compared to those in soil moisture estimates are consistent with the fact that soil moisture is only one factor determining T2m and q2m. Errors in the model parameterization of the coupling between soil moisture and screen-level parameters, for example, are not corrected through the assimilation of SMAP Tb measurements.

An examination of the OmF residuals for Tb (using SMAP observations) and atmospheric specific humidity and air temperature profiles (using radiosonde measurements) further illustrates that the benefit of the SMAP Tb analysis in the weakly coupled LADAS is not limited to soil moisture but also improves near-surface atmospheric humidity and temperature through the dynamic interactions between the land surface and the atmosphere (see Figs. 8 and 9). This result is also confirmed by a comparison of the Tb OmF residuals and soil moisture skill metrics from the LADAS with those from the land-only LDAS_offl_ experiment (see Figs. 5 and 9), which indicate slightly better skill in the LADAS compared to LDAS_offl_.

The results of this study are largely consistent with those of DR19, although it is difficult to compare their results to ours. DR19’s experimental period in 2013 precedes the launch of SMAP. Moreover, DR19 used gauge-based precipitation corrections, which increase soil moisture simulation skill in well-observed regions such as the United States and Western Europe, although they have limited impact on skill across much of the globe [29]. Compared to the MERRA-2 baseline system of DR19, however, the present study used an improved version of the GEOS ADAS, making it harder for the SMAP Tb analysis to yield improvements. Nevertheless, the assimilation of SMAP Tb observations improved the LADAS-simulated soil moisture and near surface air humidity and temperature by at least as much as did the assimilation of ASCAT and SMOS soil moisture retrievals in DR19, presumably owing to 1) the higher quality of the assimilated SMAP data and 2) the improved, spatially distributed, radiance-based Tb analysis of the L4_SM algorithm. A follow-up investigation of the separate and joint assimilation of ASCAT, SMOS, and SMAP observations in the current version of the LADAS promises to yield further insights but was beyond the scope of the present study, given the considerable computational demands of the LADAS.

The updated GEOS LADAS is, therefore, a promising tool for use in the near-real time GEOS FP weather analysis and future GEOS-based reanalysis products. Additional validation and development, however, is needed. For example, tower-based measurements of evapotranspiration and sensible heat fluxes were not yet publicly available in sufficient quantity for the JJA 2017 experimental period. Once such records become available, it is important to assess the impact of the SMAP Tb analysis on the quality of the simulated surface turbulent fluxes. Alternatively, evapotranspiration data derived from thermal infrared satellite observations could be used [87]. Moreover, the impact of the SMAP Tb analysis on medium-range weather forecasts (as opposed to the 6-h atmospheric background forecasts that are part of the atmospheric analysis) needs to be assessed.

Finally, the weakly coupled LADAS needs to be tested in the context of the quasi-operational, near-real time, 12.5-km resolution GEOS FP weather analysis, which uses a hybrid 4-D ensemble variational (Hybrid 4D-EnVar) approach [88]. Preliminary results (not shown) derived using a prototype LADAS version based on the Hybrid 4D-EnVar ADAS are encouraging, but more development is needed. The coupling of the ensemble-based LDAS subsystem to the ensemble-based atmospheric analysis involves a slew of design choices and opportunities. First, having access to an *ensemble* of surface meteorological forcing data from the Hybrid 4D-EnVar should provide a more realistic representation of uncertainty in the surface meteorological forcing of the land than do the purely statistical perturbations used heretofore in the LDAS [89]. Second, the ensemble of land increments can be fed back into the ensemble of AGCM simulations of the Hybrid 4D-EnVar ADAS (rather than feeding only the ensemble-average LDAS increments back into the ADAS). Clearly, continued development of the LADAS is needed to further constrain errors in soil moisture and near-surface atmospheric variables, thereby improving analysis estimates and medium-range forecasts of T2m, q2m, surface fluxes, and land surface conditions. More generally, the weakly coupled LADAS presented here is just one small step towards the community’s ultimate objective of a fully coupled Earth system analysis.

## Figures and Tables

**TABLE I:** Experimental Overview

Experiment name	System or subsystem; model	Analysis algorithm^a^	Assimilated observations	Surface meteorological forcing	Experiment period	Start of spin-up period	Model initial conditions:	
							Atmosphere	Land
ADAS	ADAS; AGCM	3D-Var	Operational FP data stream	ADAS AGCM (Corrector)	JJA 2017	1 Apr 2017	MERRA-2	Land model spin-up
LADAS	ADAS; AGCM	3D-Var	Operational FP data stream	LADAS AGCM (Corrector)	JJA 2017	3 May 2017	ADAS experiment	ADAS experiment
	LDAS; Catchment	EnKF (Nens=24)	SMAP Tb	LADAS AGCM (Predictor)	JJA 2017	3 May 2017	n/a	LDAS Open Loop
LDASoffl	LDAS; Catchment	EnKF (Nens=24)	SMAP Tb	ADAS (Predictor)	JJA 2017	n/a	n/a	LADAS experiment
LDAS Open Loop	LDAS; Catchment	n/a (Nens=24)	n/a	FP (Corrector)	Apr 2015–Apr 2020	1 Jan 2015 (ensemble)	n/a	Land model spin-up

ADAS and LADAS output examined in this study is from the AGCM Corrector segment.

a Number of land ensemble members is denoted with N_ens_.

## Footnotes

1 [Online]. Available: http://madis.ncep.noaa.gov
